# A comparison, for older people with diabetes, of health and health care utilisation in two different health systems on the island of Ireland

**DOI:** 10.1186/s12889-020-09529-0

**Published:** 2020-09-24

**Authors:** Tom Pierse, Luke Barry, Liam Glynn, Andrew W. Murphy, Sharon Cruise, Ciaran O’Neill

**Affiliations:** 1 Health Economic and Policy Analysis Centre, National University of Ireland Galway, Galway, Ireland; 2 Graduate Entry Medical School and Health Research Institute, University of Limerick, Limerick, Ireland; 3 Discipline of General Practice, School of Medicine, National University of Ireland Galway, Galway, Ireland; 4 Centre for Public Health, Queens University Belfast, Belfast, UK

**Keywords:** Diabetes, Complications, Health care utilisation, Quality and outcomes framework

## Abstract

**Background:**

There are social and economic differences between Northern Ireland (NI) and the Republic of Ireland (ROI). There are also differences in the health care systems in the two jurisdictions. The aims of this study are to compare health (prevalence of diabetes and related complications) and health care utilisation (general practitioner, outpatient or accident and emergency utilisation) among older people with diabetes in the NI and ROI.

**Methods:**

Large scale comparable surveys of people over 50 years of age in Northern Ireland (NICOLA, wave 1) and the Republic of Ireland (TILDA, wave 1) are used to compare people with diabetes (type I and type II) in the two jurisdictions. The combined data set comprises 1536 people with diabetes. A coarsened exact matching approach is used to compare health care utilisation among people with diabetes in NI and ROI with equivalent demographic, lifestyle and illness characteristics (age, gender, education, smoking status and self-related health, number of other chronic diseases and number of diabetic complications).

**Results:**

The overall prevalence of diabetes in the 50 to 84 years old age group is 3.4 percentage points higher in NI (11.1% in NI, 7.7% ROI, *p*-value < 0.01). The diabetic population in NI appear sicker – with more diabetic complications and more chronic illnesses. Comparing people with diabetes in the two jurisdictions with similar levels of illness we find that there are no statistically significant differences in GP, outpatient or A&E utilisation.

**Conclusion:**

Despite the proximity of NI and ROI there are substantial differences in the prevalence of diabetes and its related complications. Despite differences in the health services in the two jurisdictions the differences in health care utilisation for an equivalent cohort are small.

## Background

Diabetic care is a substantial driver of overall health care utilisation and costs. In Europe and North America, the proportion of health care expenditure on diabetes in 2010 ranges from 6 to 14% [1]. In the Republic of Ireland, the incremental cost of additional health service use is estimated to be €89 million annually [2]. Health care utilisation and health care costs among those with diabetes are strongly related to diabetic complications; in the UK, 80% of diabetic health care costs are due to complications [3, 4]. In addition to direct health care costs, diabetes significantly impacts on mortality rates, quality of life and labour market productivity [5, 6].

It is currently difficult to make direct comparisons between the prevalence of diabetes in Northern Ireland (NI) and Republic of Ireland (ROI). In Northern Ireland (NI), based on register data, 5.6% of the population aged 18 and over have diabetes [7]. While in ROI a similar register does not exist, it has been estimated that 5.2% of those aged 18 and over have diabetes [8]. However, given the strong relationship between diabetes prevalence, age and gender comparing these two figures is problematic. A previous study, based on prescribing databases in the two jurisdictions found clinically equivalent prevalence rates of diabetes in NI and ROI across all age groups [9].

NI and ROI have operate different health care systems. In both, the GP is the primary point of contact between the health service and people with diabetes. However, there are differences in the way that GP services are delivered in NI and ROI. In NI practices provide publicly funded care, free at the point of use, to a defined list of patients on a universal basis. The Quality Outcomes Framework (QOF) system, in place from 2004, provides financial incentives for GPs to maintain disease registers and meet quality indicators. The QOF system resulted in three simultaneous changes: better data collection by GPs, public information on the quality of care, and pay for performance [10]. For diabetes, GPs receive additional payment for having higher proportions of patients with biomarkers such as blood pressure, lipids and blood sugar in specified ranges as well as records of screening/examinations [11]. However, beyond the upper thresholds of each QOF indicator, GPs receive no additional financial reward to improve care [12]. In the UK QOF has been shown to be associated with improvements in both process and outcomes of diabetes care [13].

In ROI, GPs have a mix of publicly funded and private fee paying patients. The mixed nature of GP care in ROI means that GPs who see additional private patients can generate more revenue [14]. In contrast in NI, private patients as a potential revenue stream do not exist. In ROI, at the time the data for this study were collected (2011), there was no specific financial support for GPs providing primary care to patients with diabetes. While several diabetes initiatives were in place in Ireland, diabetic care was frequently unstructured and record keeping by many GPs was poor [15, 16]. However, structured reviews and record keeping are only one component of quality primary care. Access and quality of interaction in GP consultations, continuity of care, and access to practice nurses are important components of care quality [17–19]. The supply of GPs has been shown internationally to be associated with improved outcomes, such as reduced mortality [20]. In this context it is notable that there are fewer GPs in NI per capita than in ROI; the average GP list size was 1620 in NI in 2014 [21] and 1175 in ROI (1335 based on Whole Time Equivalent, WTE), based on total number (head count) of GPs for 2014 and population numbers [22]. While we do not have WTE values for GPs in NI, even if all GPs were working on a full time basis in NI, there would still be more supply in ROI. Differences in the supply of GPs may result in shorter consultation durations [23, 24] and longer waiting times for non-emergency consultations in NI, as in the rest of the UK [25, 26]. Practice nurses play an increasingly important role in the provision of primary care [27]. As with GPs there are more practice nurses per capita in ROI. In ROI there are 0.26 practice nurses per 1000, this compares with an average of 0.2 in NI [22, 28, 29].

Cost has been shown to be an important factor in the demand for GP care [30]. While GP care is free at the point of use for patients in Northern Ireland, a substantial minority (31.5%) of people in ROI with diabetes are not covered by the medical card or GP visit card schemes and will have to pay for their GP care [31]. For those who have to pay out of pocket for a GP consultation, the cost of a consultation is in the region of €50, which may represent a significant deterrent to attending [32, 33]. While the cost of attending the GP may be a deterrent for some people it may, by reducing demand, reduce capacity constraints that permit easier access for others [14].

The approach to outpatient diabetes care varies widely across public hospitals in ROI in terms of the discharging of uncomplicated cases back to primary care [34]. There is also substantial variation in waiting lists across hospitals for outpatient care. All patients have access to free public outpatient diabetes care but non-medical card holders (those who have to pay to access GP care) usually have to pay for diabetes services provided by their GP [34]. This can result in a reluctance by some patients to be discharged to their GP [34]. There is no costs to patients for attending outpatient clinics in NI. Outpatient waiting lists are not available for diabetes care.

In this study, we examine, for patients aged 50 and over with diabetes, differences between NI and ROI in the prevalence of diabetes and the number and type of health care contacts (GP, Outpatient, A&E Visits and Hospital Nights). Older people are the main population of interest for examining health care utilisation by people with diabetes given this is where the disease is most prevalent – in the UK, 83% of people with diabetes (type I and type II) are over the age of 50 [35, 36]. One previous study, using different data sources, has previously compared the age adjusted prevalence of diabetes between the two jurisdictions. Differences in entitlement to care (including sub-groups differentiated by income level) and utilization of care between the two parts of Ireland have been explored previously for the overall population [33, 37, 38]. As far at the authors are aware a needs adjusted comparison of health care utilisation by people with diabetes is not available.

## Methods

### Data

The Irish Longitudinal Study on Aging (TILDA) and Northern Irish Cohort for the Longitudinal Study of Aging (NICOLA) surveys, used in this study, are based on interviews and health assessments with a representative of the population aged 50 and over in both ROI and NI (see [39, 40] for further details of the TILDA and NICOLA studies, including design, methodology, and assessments carried out). The health assessment comprised of a physical examination and a blood test carried out by a nurse at a health centre.

The TILDA and NICOLA surveys are ideally designed for comparison with each other due to the similarity in the two surveys. Each survey consists of computer-assisted personal interviewing (CAPI) surveys containing identical questions on diabetes diagnosis and complications. Self-reported doctor diagnosed diabetes, directly comparable across the two surveys, is used in this study. The prevalence of diabetes is compared for age groups between 50 and 84 as there are no observations with diabetes over the age of 84 in the TILDA data. Self-reported health care utilisation variables examined in this study were GP utilisation, outpatient, A&E and hospital nights. Utilisation rates are all based on the previous 12 months. Survey work for TILDA was carried out in 2011 (Wave 1), survey work for NICOLA was carried out in 2014–2016 (Wave 1). Using the first wave of each survey is advantageous as attrition in later waves may not be at random. Estimates of the number of cases of diabetes in NI are based on NICOLA age and gender adusted prevalence rates and population estimates for 2014 [41].

### Statistical approach

Matching is a method for rebalancing observational data [42]. A key advantage of matching over regression analysis is a reduced dependence on assumptions about functional form [43]. A Coarsened Exact Matching (CEM) approach is used here to match observations in the two jurisdictions and allow for a direct comparison of outcomes. CEM is a type of matching process that involves the temporary coarsening of continuous variables followed by a direct matching between two groups. The CEM method involves categorising continuous variables into user defined groups. A stratum is created for each unique observation in the data set. Observations are dropped that do not have at least one observation from the two groups in the stratum. Thus, the CEM process involves pruning from both groups [44]. This method was chosen over the Mahalanobis distance method of matching as we wish to match on a combination of continuous and dichotomous variables [45].

### Matching variables

Matching is done based on the presence and severity of health needs. Health needs are captured in age, sex, education, current smoking status, self-reported health, the number of chronic illnesses and the number of diabetic complications reported. Diabetic complications are defined here as heart attack, stroke, leg ulcers, kidney disease, neuropathy, retinopathy and nephropathy. These matching variables were selected based on previous findings from TILDA of the covariates of health care utilisation [46]. A count of the number of diabetic complications is included as this has been shown to correlate with hospital utilisation [47]. Similarly, chronic illnesses identified in the surveys were included in the matching as a count as there is little indication that more complex multi-morbidity indexes outperform a simple count [48]. The available chronic illness in the surveys are: chronic lung disease; asthma; arthritis; osteoporosis; cancer; any emotional, nervous or psychiatric problems; alcohol or substance abuse; stomach ulcers; varicose ulcers; high blood pressure or hypertension and angina. Body Mass Index (BMI), glycosylated haemoglobin (HbA1c) and other biometric markers are not included in the analysis of health care utilisation as they are only available for the subsample that attended the health assessment.

### Number of observations

In total 17,008 people were surveyed (TILDA: 8504 and NICOLA: 8504). Both studies contain a small number of observations under 50 years included in the matching process (TILDA: 329 and NICOLA: 195). There were 16,681 observations (TILDA: 8468 and NICOLA: 8212) with no missing data in the matching and outcome variables. The combined data set comprises complete surveys from 1536 people with diabetes (TILDA: 634 and NICOLA: 902). After the matching process there are a total of 906 observations (TILDA: 420 and NICOLA: 486).

## Results

### Prevalence of diabetes

**Fig. 1:**
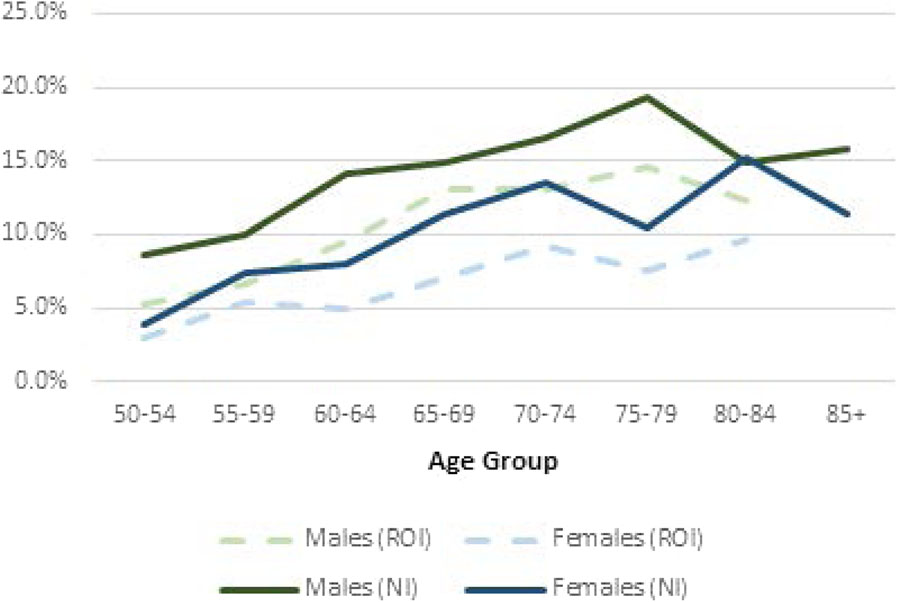
Prevalence of diabetes by gender and age category

Figure 1 shows the percentage of individuals who report having received a diagnosis of diabetes from their doctor by sex and age group in NI and ROI. The prevalence rates for NI are higher across all age groups and genders. The overall prevalence in the 50 to 84 years old age group is 3.4 percentage points higher in NI (11.1% in NI, 7.7% ROI, *p*-value < 0.01). There are no observations in ROI for those in the over 85 years age group.

There is no indication from the data available that the higher prevalence rates in NI are due to higher case ascertainment. Among those for whom HbA1c data were available, the rates of undiagnosed diabetes (HbA1c > 48 mmol/mol (6.5%) and no report of a diabetes diagnosis) are also higher in NI (0.8% in ROI vs 4.4% in NI).

### Health of people with diabetes

Table 1 shows the demographic and health status of the samples of people with diabetes in ROI and NI. There are no differences (*p* > 0.05) in the gender and current smoking status of the two samples, the sample from NI is slightly older. As previously reported, people in the NI have significantly lower levels of self-reported good health status and higher rates of education [49]. People with diabetes in NI have substantially more complications related to diabetes; the proportion of people with diabetes and two or more complications is 8.9 percentage points higher (*p* < 0.001) in NI. Rates of stroke, kidney disease and neuropathy are all significantly higher in NI. The number of other chronic diseases that people with diabetes have is also higher in NI; the proportion of people with diabetes, with three or more other chronic conditions is 9.4 percentage points higher (*p* < 0.001) in NI. Chronic lung disease, asthma, arthritis, alcohol or substance abuse, emotional, nervous or psychiatric problems, hypertension and angina are all significantly higher in NI (*p* < 0.05). In summary, the sample of people with diabetes in NI appear substantially “sicker” than the sample from ROI.

**Table 1:** Health and demogrpahic characterisitcs of older people with diabetes in ROI and NI (before matching)

	ROI (TILDA)		NI (NICOLA)		***p***-value
	n		n		
Number of People with Diabetes	634		902		
Age (Mean)		66.4		67.5	0.018
Male	365	58%	501	56%	0.430
Primary Education Only	260	41%	314	35%	0.014
Current Smoker	109	17%	148	16%	0.685
Good Self Related Health	320	50%	345	38%	< 0.001
*Diabetic Complications*					
None	403	64%	482	53%	< 0.001
One	167	26%	249	28%	0.58
Two +	64	10%	171	19%	< 0.001
Heart Attack	73	11.5%	127	14.1%	0.142
Stroke	40	6.3%	87	9.6%	0.020
Leg ulcers	24	3.8%	39	4.3%	0.601
Kidney disease	41	6.5%	129	14.3%	< 0.001
Neuropathy (Nerve Endings)	90	14.2%	173	19.2%	0.011
Retinopathy	47	7.4%	83	9.2%	0.215
Nephropathy (Kidney)	23	3.6%	74	8.2%	< 0.001
*Treatment*					
Tablets	498	78.5%	678	75.2%	0.124
Insulin	107	16.9%	185	20.5%	0.074
*Other Chronic diseases*					
None	113	18%	108	12%	0.001
One	215	34%	280	31%	0.230
Two	188	30%	261	29%	0.760
Three+	118	19%	253	28%	< 0.001
Chronic lung disease	30	4.7%	77	8.5%	0.004
Asthma	72	11.4%	134	14.9%	0.048
Arthritis	204	32.2%	382	42.4%	< 0.001
Osteoporosis	34	5.4%	62	6.9%	0.229
Cancer	46	7.3%	84	9.3%	0.154
Any emotional, nervous or psychiatric problems	63	9.9%	142	15.7%	0.001
Alcohol or substance abuse	14	2.2%	37	4.1%	0.042
Stomach ulcers	47	7.4%	10	1.1%	< 0.001
Varicose Ulcers	24	3.8%	53	5.9%	0.065
High blood pressure or hypertension	390	61.5%	602	66.7%	0.004
Angina	72	11.4%	143	15.9%	0.048

### Health care utilisation

Table 2 shows the health care utilisation of people with diabetes in NI and ROI before and after matching. Matching is carried out based on age, sex, education, smoking status, self-related health, number of diabetic complications and number of chronic illnesses. The left hand side of Table 2 shows the health care utilisation of people with diabetes in both jurisdictions before matching. There is no statistical difference between the GP utilisation but outpatient visits; A&E visits and Hospital nights are all significantly higher in NI. Interestingly substantially more people in NI with diabetes reported not having attended their GP in the last year (ROI 4.2%, NI 7.9%, *p*-value = 0.006). A similar proportion of people with diabetes attended an outpatient service in the past 12 months (ROI 38.9%, NI 38.2%, p-value = 0.79).

**Table 2:** Health care utilisation in the last 12 months by older people with diabetes

	Before Matching		***p***-value	After Matching		***p***-value
	ROI (TILDA)	NI (NICOLA)		ROI (TILDA)	NI (NICOLA)	
No. of people with diabetes	634	902		420	486	
GP visits per year (mean including non-users)	5.7	5.6	0.668	5.6	5.0	0.108
GP visits: None	4.2%	7.9%	0.004	4.5%	9.3%	0.006
Outpatient visits per year (mean including non-users)	2.1	3.6	< 0.001	2.1	3.2	0.079
Outpatient Visits: None	38.9%	38.2%	0.79	40.0%	42.4%	0.47
A&E visits per year (mean including non-users)	0.3	0.5	0.010	0.3	0.4	0.290
Hospital nights per year (mean including non-users)	1.2	2.1	< 0.00	1.2	1.8	0.039

The right hand side of Table 2 shows the health care utilisation of people with diabetes in both jurisdictions after the matching process. The health care utilisation of the NI group reduced in all areas of utilisation – this is what you would expect as higher needs observations in NI are pruned off in the matching process. After matching for need, the difference between primary care utilisation in ROI and NI is now larger, but still not statistically significant (ROI 5.6, NI 5.0, *p*-value = 0.108). After matching for need, the difference between secondary care utilisation in ROI and NI is reduced, and in most cases cease to be statistically significant. In the case of inpatient nights however, NI patients continue to consume significantly more care.

## Discussion

In this study we compare the prevalence, health and patterns of health care usage of people with diabetes in Northern Ireland and the Republic of Ireland based on two samples taken from representative cohort studies in the respective jurisdictions.

The surveys show that on an age and gender adjusted basis there is a substantially higher prevalence of diabetes in NI. This contrasts with the previous finding of clinically equivalent prevalence rates of diabetes in NI and ROI across all age groups [9]. The main difference between this study and the previous study [9] is in the NI prevalence rates. The rates of diabetes shown here for ROI are in line with previous studies for ROI [9, 50, 51]. The rates of diabetes shown in the NICOLA data are consistent with the total number of cases of diabetes registered in Northern Ireland [7]. The estimated number of people over 50 with diabetes based on the NICOLA prevalence rates was 67,941 in 2014. This represents 83% of the 81,867 people in NI with a diagnosis of diabetes in the same year on the diabetes register. This is in line with the proportion of people with diabetes who are over 50 in the rest of the UK [35, 36]. Our results also show that among people with diabetes, those in NI have more complications and more chronic illnesses in general, than people with diabetes in ROI.

While it is possible that having a system like QOF may have increased detection and diagnosis of certain conditions, leading to greater prevalence in NI, this is not supported by the lower self-reported health and GP utilisation in the North. Therefore we believe it is not likely that differences in the prevalence of diabetes, diabetic complications and chronic illness can be explained by the differences in the healthcare system between the two jurisdictions. We also find no indication from the data available in the surveys that the higher prevalence rates in NI are due to higher case ascertainment. While alternative data sources for the rates of undiagnosed diabetes in ROI show substantially higher rates compared to TILDA they are still lower than in NI [52].

Broader societal factors, that may include attitudes to diet and physical exercise, other diseases or possibly cumulative lifetime stress may also contribute to the difference in the prevalence rates. There is limited data available that allows for the direct comparison between the two jurisdictions of health indicators relating to diabetes [53, 54]. From the available data it is not possible to point towards a likely causal mechanism as to why the North would have more diabetes – for example the prevalence of obesity, levels of physical activity and smoking are similar in the two jurisdictions [53]. Chronic stress, related to the protracted period of civil unrest known as the “Troubles” may be one potential explanation for differences [55] though this is speculative.

People with diabetes typically have other chronic conditions [56]. By adjusting for the “sickness” of the people with diabetes in NI and ROI through matching we show that health care utilisation with the exception of inpatient care is similar in both jurisdictions despite differences in health care systems. While not statistically significant, the results point towards a more primary care focused service in ROI, with more frequent GP visits and less frequent secondary care visits. A previous comparison between the two jurisdiction of health care utilisation in general similarly found a greater role played by GPs in ROI [57].

Policies such as QOF and universal access to GP care in NI might be expected to increase health care utilisation in NI, however, this appears not to be the case. While direct financial incentives were not in place at the time in ROI for diabetes care, the higher levels of primary care utilisation seen may relate to the greater availability of GPs in ROI where there are substantially more GPs per capita [21, 22]. The suite of policies required to improve the supply of GPs goes beyond a narrow set of financial incentives, including increasing GP training intake [22]. The Cycle of Care policy was introduced in ROI in 2015, subsequent to the TILDA data used in this paper. This policy incentivises GPs to provide structured annual reviews and improved records for people with type 2 diabetes. While this may have improved patient care in ROI [58] any decline in the availability of GPs per capita in the future due to increased demand [59], or supply factors such as training emigration or retirements [22] may counteract any positive benefits in the long run. In addition, the Cycle of Care scheme does not provide funding for patients without public medical cover, which comprise 32% of people over 50 years of age with diabetes [31].

It is also notable there is a higher prevalence among people with diabetes in NI of non-attendance at the GP in the past 12 months. As the cut points (proportion of people with diabetes on a practices register) for payments, however, are currently set there are no financial incentives to seek out the people who do not attend once the practice reaches a certain cut off point. This may contribute to poorer disease management and in part explain the greater use of hospital services in Northern Ireland. Higher levels of hospital inpatient utilisation in Northern Ireland may also be due to higher levels of hospital supply [60].

### Limitations

The data used in this study are based on two large representative cross sectional surveys. However there are a number of limitations to our study. The presence of diabetes, complications, and health care utilisation are all self-reported, running the risk of recall bias. However, there is no reason to believe that recall bias would apply differentially across jurisdictions. The study compares aggregate differences in health care utilisation among those with diabetes and not differences between sub-groups among whom entitlements might differ. While this may provide a fruitful avenue for future research, it was considered to be beyond the scope of this current work. The study considers type 1 and type 2 diabetes together. However the number of people with type 1 diabetes in the surveys is likely to be very small – in the UK less that 4% of people over 50 with diabetes are type 1 [35].

The age and gender adjusted prevalence rates of diabetes and diabetic complications are compared at two time points over 3 years apart; a comparison of these rates on the same year may reduce or increase the scale of the differences depending on the relative difference in the incidence of diabetes and complications in that 3 year period. A comparison cannot be made between people with diabetes in the over 85 year old age group as none are present in the TILDA data set. People with dementia are similarly underrepresented in both TILDA and NICOLA. The severity of reported health conditions is not available in the data; the variation in health care needs may not be fully captured by the number of chronic illnesses or the number of diabetic complications. Only key aspects of health care utilisation (general practitioner, outpatient or accident and emergency utilisation) are compared in this study, other primary and social care utilisation are not compared.

## Conclusion

This study shows that the prevalence and severity of diabetes, among those aged 50 and over, is higher in Northern Ireland than in the Republic of Ireland. The study shows that for cohorts with comparable health care needs, with the exception of inpatients nights, there is no significant difference in patterns of health care use. The available data does not allow us to explain the underlining reasons for differences in health care utilisation across the two jurisdictions. For GP utilisation, there are a range of factors including the supply of GPs and financial incentives of both GPs and patients that will affect utilisation. The lack of difference in GP utilisation, despite greater severity, combined with higher rates of GP non-attendance in NI suggest a closer examination of primary care is worthy of investigation. However, it is useful for policy making to know that a substantially different health care system can produce a very similar pattern of utilisation.

## Data Availability

The data that support the findings of this study are available on request from TILDA (https://www.ucd.ie/issda/data/tilda/) and NICOLA (https://www.qub.ac.uk/sites/NICOLA/).

## Abbreviations

BMI: Body Mass Index
CEM: Coarsened Exact Matching
GP: General practitioner
QOF: Quality and Outcomes framework
NI: Northern Ireland
ROI: Republic of Ireland
